# Design and Electrical Performance Simulation of a Novel 3D Silicon Detector with Interleaved Trench Electrodes

**DOI:** 10.3390/mi17090993

**Published:** 2026-08-23

**Authors:** Xuyang Song, Tao Long, Jun Zhao, Chunxiang Ni, Xinqing Li, Manwen Liu, Kun Wang, Jiahao Fu, Taiping Lu, Zheng Li

**Affiliations:** 1 Shandong Provincial Engineering Research Center for Optoelectronic Sensing Materials and Device Micro-Nano Manufacturing, School of Integrated Circuits, Ludong University, Yantai 264025, China; 16634270575@163.com (X.S.); zhaojun@ldu.edu.cn (J.Z.); 13465506066@163.com (C.N.); uukkk@163.com (K.W.); m15169874527@163.com (J.F.); taipinglu@ldu.edu.cn (T.L.); 2 Engineering Research Center of Photo Detector Special Chip in Universities of Shandong, Ludong University, Yantai 264025, China; xqli1996zz@163.com; 3 School of Materials Science and Engineering, Xiangtan University, Xiangtan 411105, China; 4 Institute of Microelectronics, Chinese Academy of Sciences, Beijing 100029, China; liumanwen@ime.ac.cn

**Keywords:** 3D silicon detector, interleaved trench electrode, TCAD simulation, charge collection efficiency, low-field region reduction

## Abstract

To address the limitations of traditional trench electrode silicon detectors, such as charge collection dead zones and non-uniform electric fields, this paper proposes a novel 3D silicon detector based on an interleaved trench electrode structure. By constructing interleaved N-type and P-type heavily doped trenches on a lightly doped N-type substrate, enhanced uniformity and depth profile optimization of the electric field distribution are achieved. Moreover, 3D numerical simulations based on TCAD demonstrate that the proposed structure provides a uniform, planar-like electric field distribution; reduces low-field regions; and enables fast carrier collection with a low full-depletion voltage. The results provide a theoretical basis and design reference for the development of novel 3D radiation detectors with low depletion voltages, fast charge collection, and favorable radiation response characteristics.

## 1. Introduction

Silicon detectors are core sensing components in high-energy physics experiments [1,2,3], astrophysics observations [4,5], nuclear radiation monitoring [6,7,8,9], and medical imaging [10,11,12]. Their performance directly affects the reliability of high-precision detection systems. With the continuous upgrading of energy levels in particle physics experiments and the increasing complexity of space exploration missions, the limitations of traditional 2D silicon detectors (such as silicon micro-strip detectors, silicon pixel detectors, and silicon drift detectors) in strong radiation environments have rendered them increasingly inadequate; this is mainly manifested as insufficient radiation damage tolerance, degradation in charge collection efficiency, and limited signal response speeds [13,14,15,16].

In 1997, the first-generation 3D columnar electrode detector [17] was pioneered by S. Parker’s team, who achieved a through-electrode structure by etching vertical holes in the silicon substrate and filling them with doped polysilicon to form columnar electrodes. However, this structure has a significant flaw: the electric field strength near the electrodes is excessively high, while the electric field in the central symmetric region of the electrodes is relatively weak, resulting in a significantly non-uniform electric field distribution. This non-uniformity leads to a sharp decline in charge collection efficiency in high-radiation environments, limiting its application in extreme environments such as the Large Hadron Collider. In 2009, Brookhaven National Laboratory in the United States proposed the 3D trench electrode silicon detector [18], marking the birth of the second generation of 3D silicon detectors. This technology utilizes a trench etching process to form continuous trenches around the detection unit, and, after heavy doping on the trench walls, polysilicon is filled to form electrodes. Compared with the columnar electrode structure, the trench electrode structure allows the electric field to be distributed more uniformly within the detection unit, mitigating the presence of low-electric-field regions. The electrical crosstalk between detection units is also significantly reduced, which is beneficial for high-density array integration. Furthermore, the drift paths of charge carriers in a uniform electric field are shortened, reducing the probability of them being trapped by radiation-induced defects. However, traditional 3D trench electrode silicon detectors still face certain challenges in practical applications. First, to maintain the mechanical integrity of the detector, trench electrodes are often left non-penetrating, which leads to the formation of “dead zones”, where the electric field strength is close to zero at the bottom of the trench or at specific geometric corners. The charge collection speed in these areas is extremely slow, and carrier recombination trapping is highly likely to occur, thereby limiting the overall charge collection and response time of the detector. Second, although the electric field distribution is superior to that in the columnar structure, electric field distortion still exists at the electrode edges and the supporting substrate boundaries, making it difficult to achieve ideal global uniformity.

To address the above issues, this paper proposes a novel 3D silicon detector structure with interleaved trench electrodes. Through the interleaved arrangement of heavily doped N-type and P-type trench electrodes, the proposed design has the following advantages over the conventional 3D pixel detector: (1) it eliminates the shortcomings of the traditional one, where the pixel size is limited by the electrode spacing (with a large pixel size, small electrode spacing is achieved here); (2) it produces a planar-like lateral electric field; and (3) it reduces the low-field regions associated with conventional non-penetrating trench structures and improves the electric field uniformity within the detector unit.

## 2. Structural Design of the 3D Silicon Detector

### 2.1. Reference 3D Trench Electrode Detector

**Figure 1:**
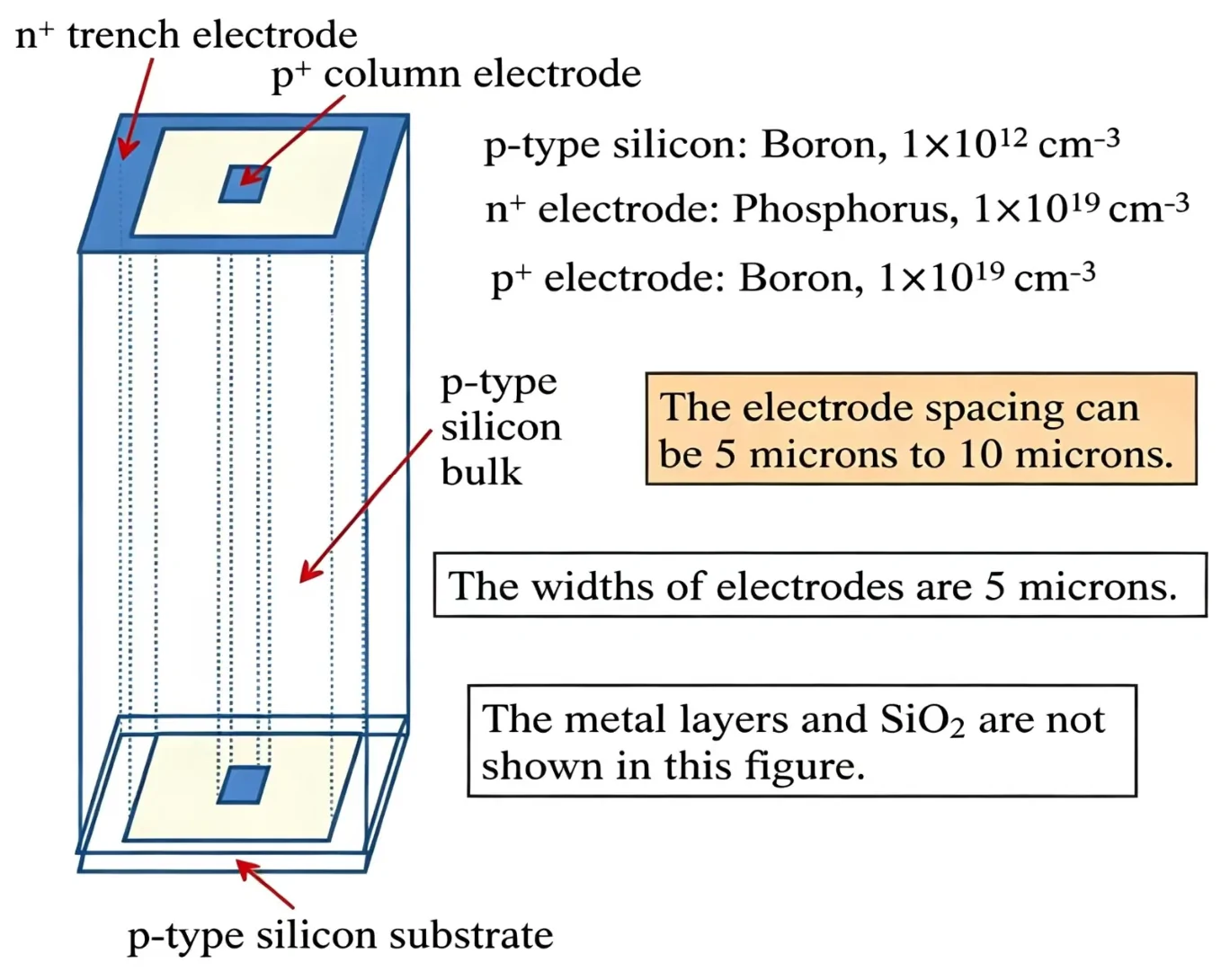
Schematic diagram of the reference ultra-fast 3D trench electrode detector [19].

The ultra-fast 3D trench electrode silicon detector reported by Liu et al. [19] is selected as the reference structure in this work. As shown in Figure 1, the detector consists of a central p+ column electrode surrounded by an n+ trench electrode in a lightly doped p-type silicon substrate. The three-dimensional electrode configuration shortens the lateral carrier drift distance and enables a low depletion voltage and fast signal response.

However, the trench electrode does not completely penetrate the silicon substrate, leaving an unetched silicon layer at the bottom for mechanical support. This region has a relatively weak electric field and may therefore form a low-field region that is unfavorable for efficient carrier transport. Therefore, further optimization of the electrode termination and supporting silicon geometry remains possible. These limitations motivate the interleaved trench electrode structure proposed in the following section.

### 2.2. Structural Design of the Novel 3D Silicon Detector with Interleaved Trench Electrodes

**Figure 2:**
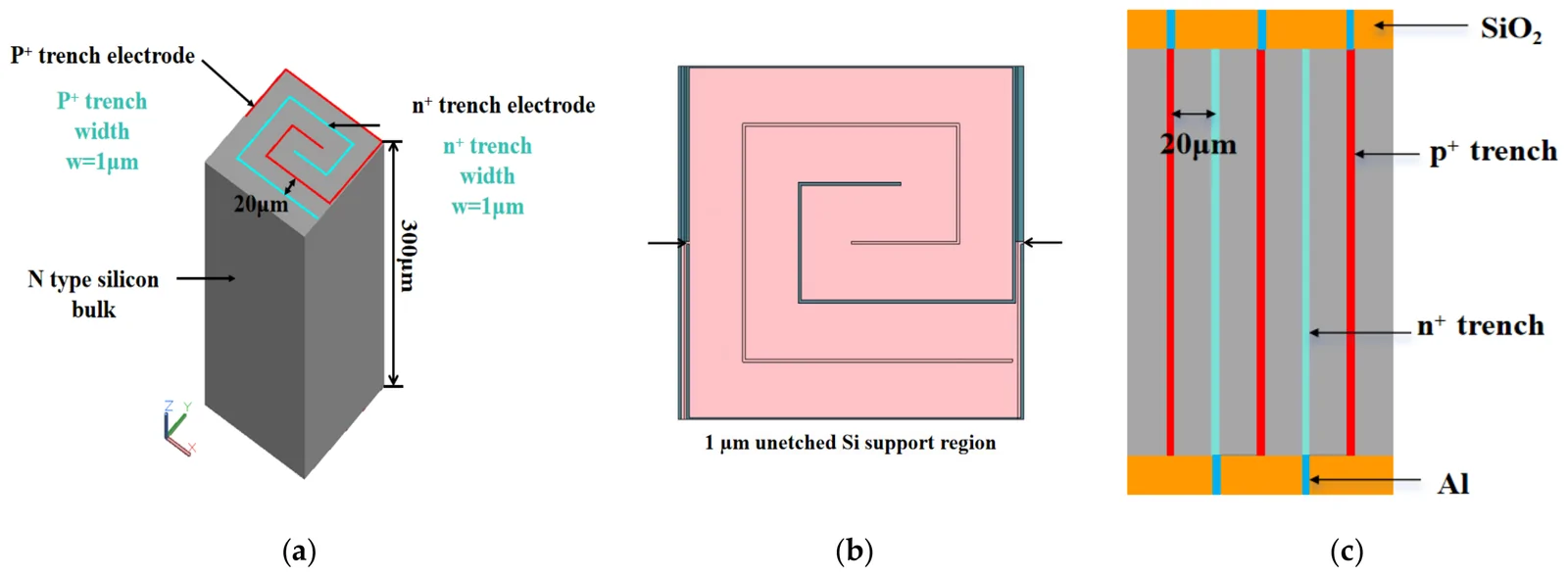
Schematic diagram of the detector structure: (**a**) 3D overall structure; (**b**) top view with 1 µm unetched Si support regions on both lateral sides; (**c**) cross-sectional view.

In contrast to the reference detector described in Section 2.1, the proposed detector employs interleaved n+ and p+ trench electrodes. As shown in Figure 2, a square geometry with a size of 120 × 120 µm^2^ and a thickness of 300 µm is selected as the detector unit. The bulk is an N-type lightly doped substrate with a doping concentration of 1 × 10^12^ cm^−3^; N-type heavily doped trenches with a doping concentration of 1 × 10^19^ cm^−3^ and P-type heavily doped trenches with a doping concentration of 1 × 10^19^ cm^−3^ are interleaved and distributed. The spacing between the two types of trenches is 20 µm, and the width of the trenches is 1 µm (Figure 2a). To maintain the mechanical integrity of the detector during trench etching, an approximately 1 μm wide unetched silicon support region is intentionally retained on each lateral side of the detector (Figure 2b). These two thin silicon regions mechanically connect the detector body to the surrounding substrate and prevent the silicon bulk from detaching during fabrication. A 1 μm thick aluminum contact is formed on the n+ and p+ trench regions, respectively. In the regions without metal contact, a 1 μm thick SiO_2_ layer is deposited for surface passivation and lithographic masking (Figure 2c) [20]. The proposed 300 μm thick detector with approximately 1 μm wide trenches represents a challenging high-aspect-ratio geometry. Its fabrication would require careful control of the trench depth, sidewall profile, corner rounding, dopant diffusion, metallization, and mechanical stability. The 1 μm wide lateral silicon support regions are intentionally retained to improve mechanical robustness during processing. Detailed process tolerance and geometrical parameter sensitivity studies will be carried out in future work.

## 3. Electrical Performance Simulation and Analysis

**Figure 3:**
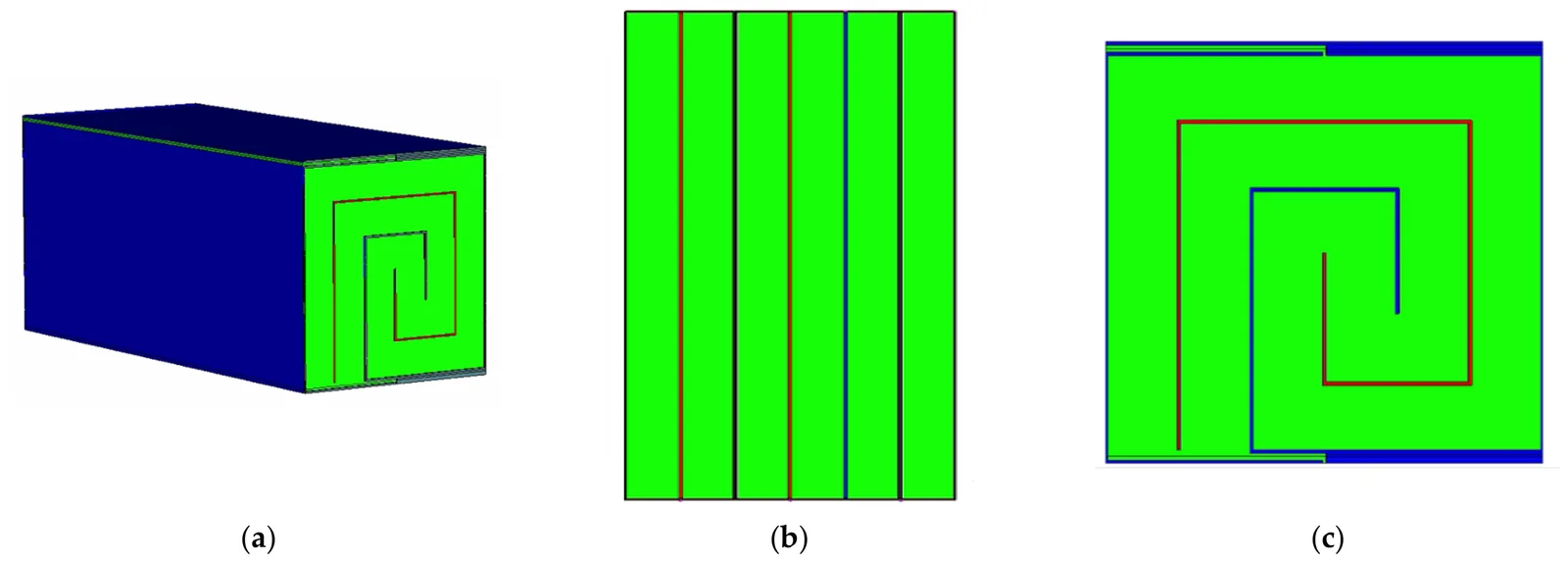
A 3D simulation schematic diagram of the detector: (**a**) overall structure; (**b**) cross-sectional view; (**c**) top view.

In this section, we present simulation results obtained utilizing Sentaurus TCAD [21,22,23] for simulation research. Sentaurus TCAD is an industry-recognized and widely used semiconductor process and device numerical simulation software platform. Based on the finite element and finite volume numerical calculation methods, this platform can accurately simulate the microscopic physical mechanisms inside semiconductor devices by self-consistently solving Poisson’s equation, carrier continuity equations, and various advanced physical transport models (such as drift diffusion models, thermodynamic models, etc.). When simulating the electrical performance of devices, Sentaurus can not only support the construction of complex 2D or 3D geometric topologies but also accurately calculate and visualize the internal electric field distribution, electric potential profiles, and carrier transport characteristics of the device under different bias conditions. Through this simulation method driven by underlying physical models, researchers can intuitively reveal the influence rules of structural parameters such as the geometric dimensions and doping distribution on the macroscopic characteristics of the device, thereby effectively predicting and optimizing the device’s performance before actual fabrication, significantly reducing the research and development costs and trial-and-error cycles. Figure 3 displays the 3D model of the novel interleaved trench electrode 3D silicon detector in the TCAD simulation. To model radiation-induced bulk damage at fluences of 1 × 10^15^ n_eq_/cm^2^ and 1 × 10^16^ n_eq_/cm^2^, a three-level defect model consisting of two acceptor traps and one donor trap was implemented in Sentaurus TCAD based on the radiation damage model reported by Moscatelli et al. [24]. The corresponding trap energy levels were E_C_ −0.42, E_C_ −0.46 eV, and E_V_ +0.36 eV, respectively. Carrier capture cross-sections were in the range of 10^−15^ to 10^−14^ cm^2^, and the radiation-induced defect concentrations were scaled with neutron-equivalent fluence.

### 3.1. Electrostatic Potential Distribution

**Figure 4:**
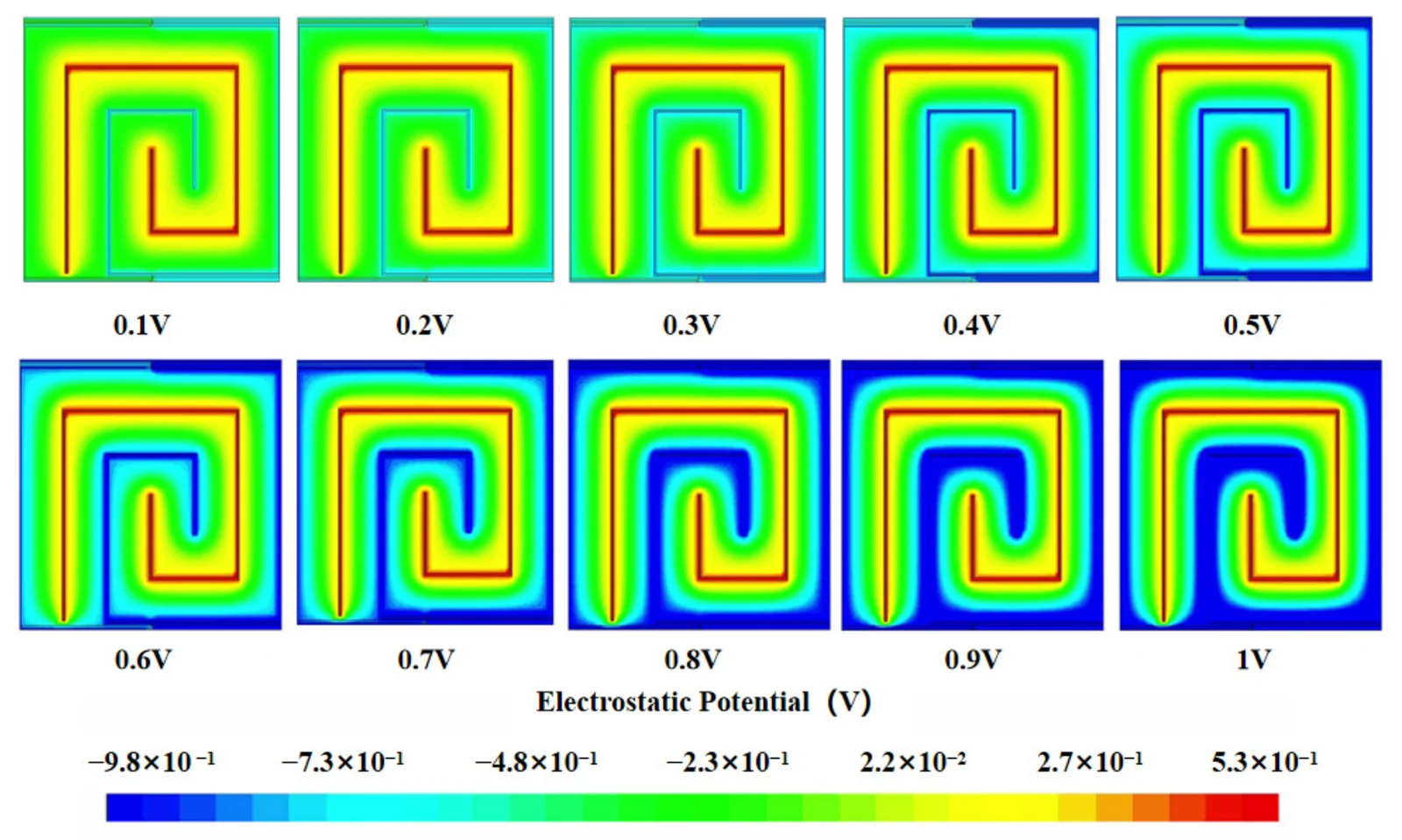
The 2D electrostatic potential distribution of the detector under different bias voltages.

Figure 4 illustrates the evolution of the internal electrostatic potential distribution of the device on the Z = 150 µm cross-section under bias voltages from 0.1 V to 1 V. The simulation results show that the potential distribution exhibits a high degree of conformality with the interleaved spiral trench structure. With an increase in bias voltage, the high-potential region shows an obvious expansion trend. The electric potential distribution remains highly uniform along the trench sidewalls.

### 3.2. Electric Field Distribution

**Figure 5:**
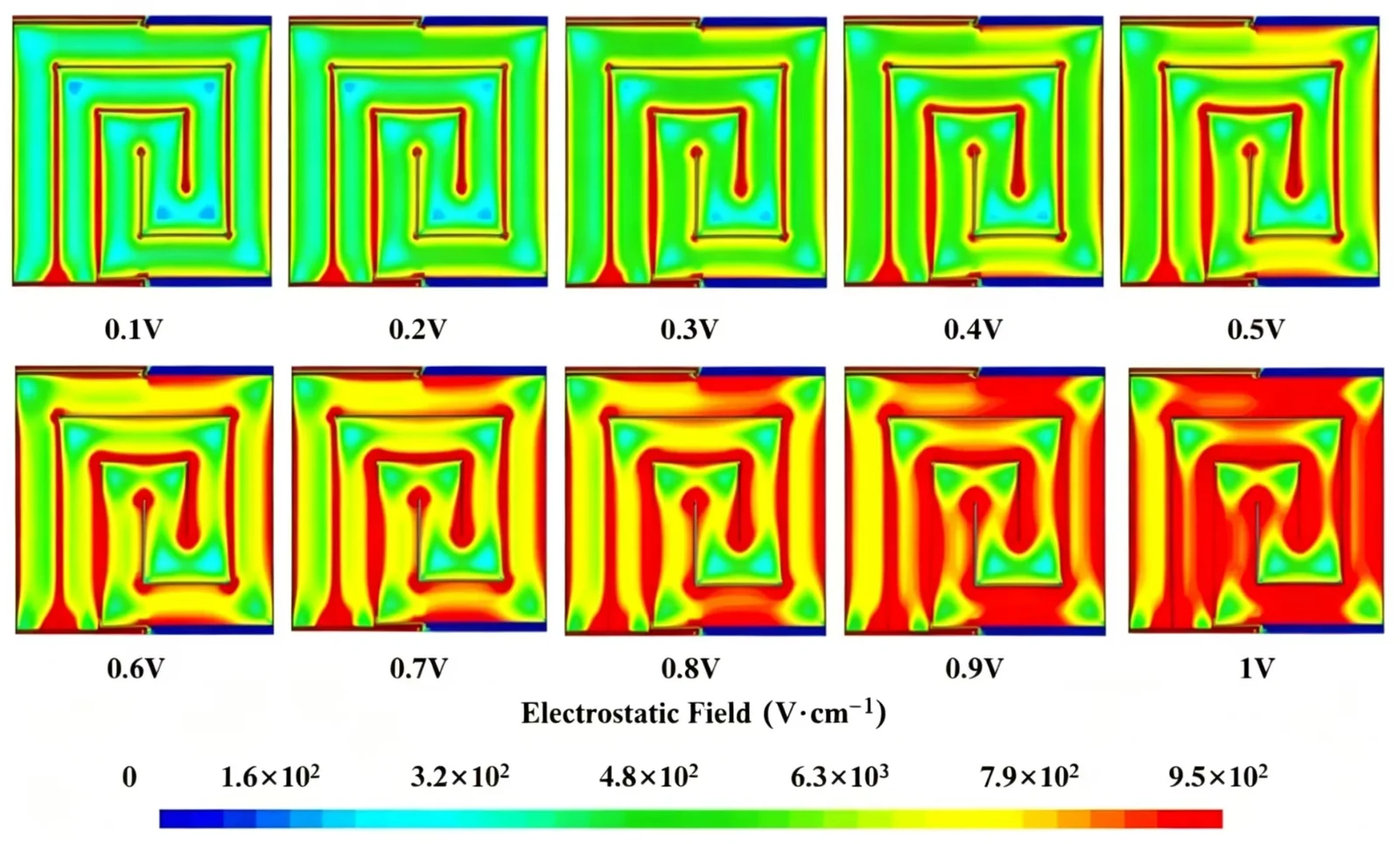
The 2D electric field distribution of the detector under different bias voltages.

Figure 5 shows the 2D electric field distribution inside the device on the Z = 150 µm cross-section under bias voltages from 0.1 V to 1 V. The simulation results demonstrate that, as the bias voltage increases, the internal electric field strength of the detector gradually intensifies, and the electric field distribution tends to be uniform. Under a bias of 0.3 V, the minimum internal electric field strength on the investigated Z = 150 μm mid-plane reaches approximately 200 V/cm. When the bias voltage increases to 0.7 V, the electric field distribution on the investigated Z = 150 μm mid-plane becomes more uniform, and the low-field region on this plane is substantially reduced.

### 3.3. Electron Concentration Distribution

**Figure 6:**
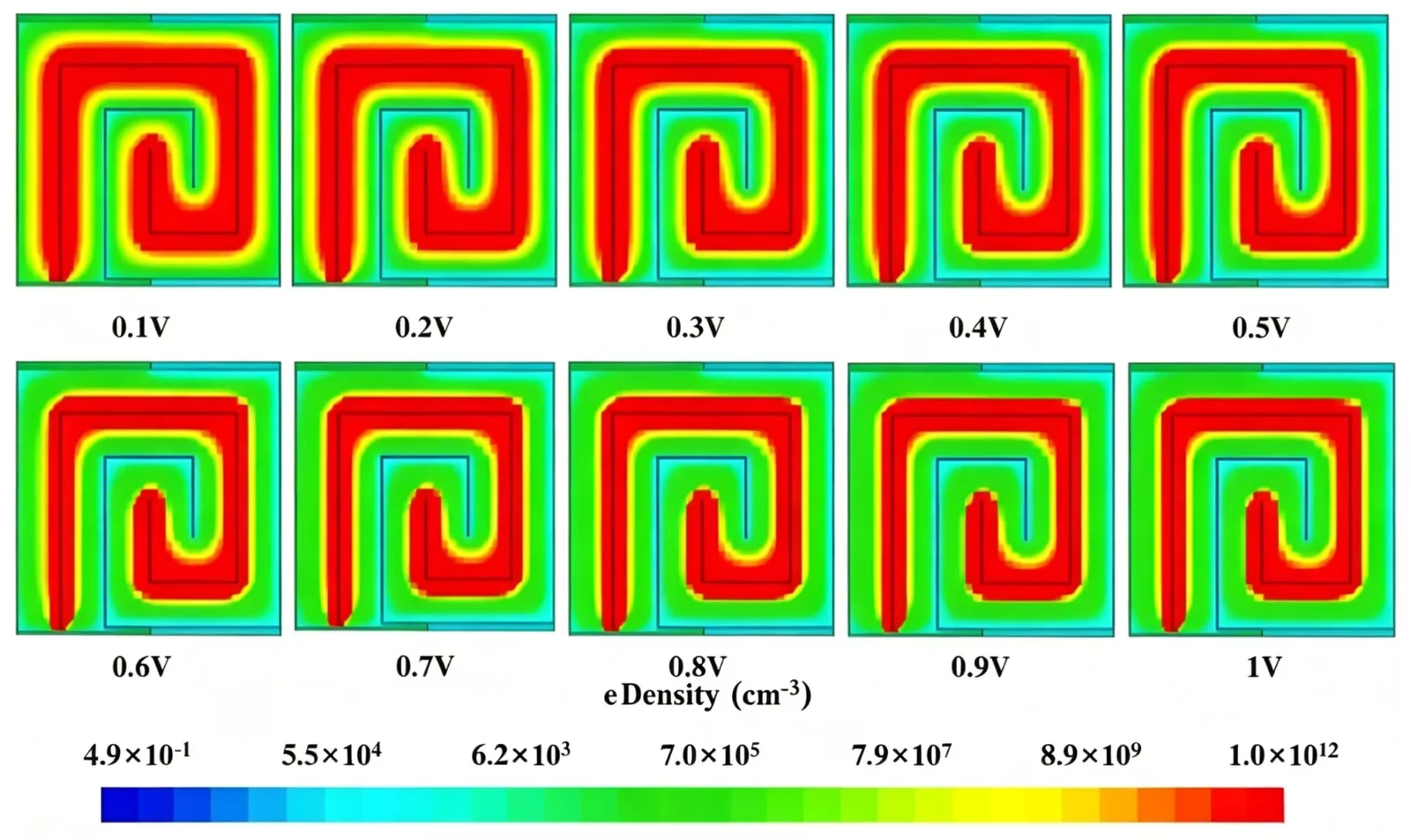
The 2D electron concentration distribution of the detector under different bias voltages.

Figure 6 displays the spatial distribution evolution of the electron concentration inside the detector on the Z = 150 µm under different bias voltages. It can be observed that there are significant differences in the spatial distribution of the electron concentration: the concentration near the cathode trench region is relatively low, while high concentration accumulation is maintained around the collecting anode trench. With a gradual increase in the bias voltage, the overall electron concentration in the device body shows a continuous downward trend. It is worth noting that, when the bias voltage reaches 0.7 V, the electron concentration in the active area drops to one-to-two orders of magnitude lower than the initial doping concentration. If the bias is further increased thereafter, the electron concentration tends to be stable and remains at an extremely low level. This characteristic clearly indicates that the detector has reached a fully depleted state at this time.

**Figure 7:**
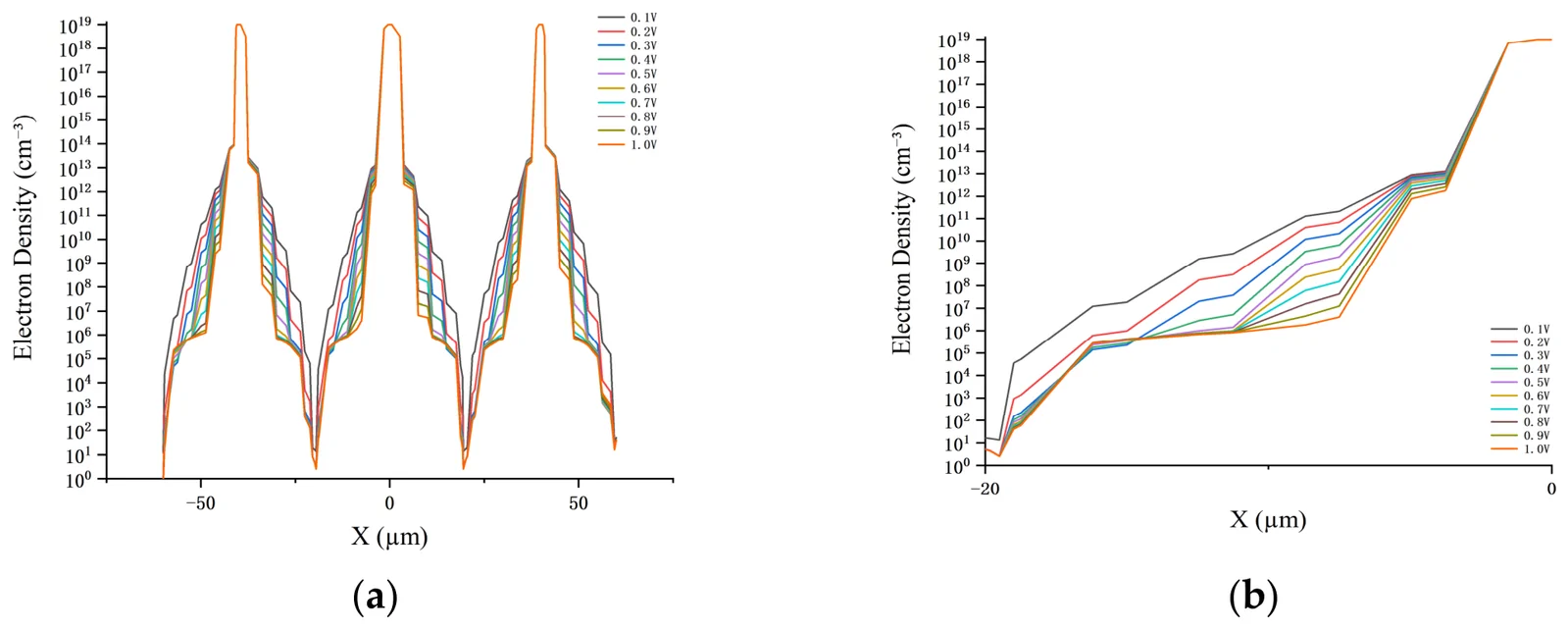
The 1D electron concentration profiles of the detector under different bias voltages: (**a**) overall profile; (**b**) local enlarged profile.

Figure 7 shows the 1D electron concentration distribution profile of the detector along the *X*-axis under different bias voltages (0.1 V to 1.0 V). Figure 7a is the overall distribution profile, and Figure 7b is the local enlarged view near the anode electrode edge region. From Figure 7a, it can be clearly observed that there are three peak regions with electron concentrations as high as 10^19^ cm^−3^ in space, corresponding to the N-type heavily doped regions (e.g., collecting anode trenches) inside the detector. Between these heavily doped regions is the active bulk region of the detector (i.e., the “valley” bottom of the curve). As the reverse bias voltage gradually increases from 0.1 V, a large number of electrons in the bulk region are depleted, leading to a sharp drop in the electron concentration of several orders of magnitude, visually reflecting the lateral expansion process of the depletion region in the bulk. Figure 7b further reveals the transition details between the high-concentration electrode region and the low-concentration bulk region. In this region, the electron concentration along the *X*-axis exhibits a significant stepped distribution characteristic towards the anode trench electrode. As the bias continues to rise, the electron concentration on the bulk region side (around X-coordinates −20 µm to −10 µm) is continuously suppressed. Notably, when the bias voltage reaches 0.7 V and above, the electron concentration curves at the bottom begin to overlap and tend to saturate. This indicates that, under biases higher than 0.7 V, the carriers in the detector bulk are fully depleted, bringing the device into a fully depleted operating state.

### 3.4. Capacitance Characteristics

Capacitance–voltage (C-V) simulations were performed to determine the full-depletion voltage of the proposed detector and to investigate the evolution of its capacitance before and after irradiation. The full-depletion voltage was extracted from the C-V characteristics [25] using the double-tangent intersection method. The small-signal AC analysis was conducted at a frequency of 1 MHz with an AC amplitude of 10 mV applied between the n^+^ and p^+^ trench electrodes.

**Figure 8:**
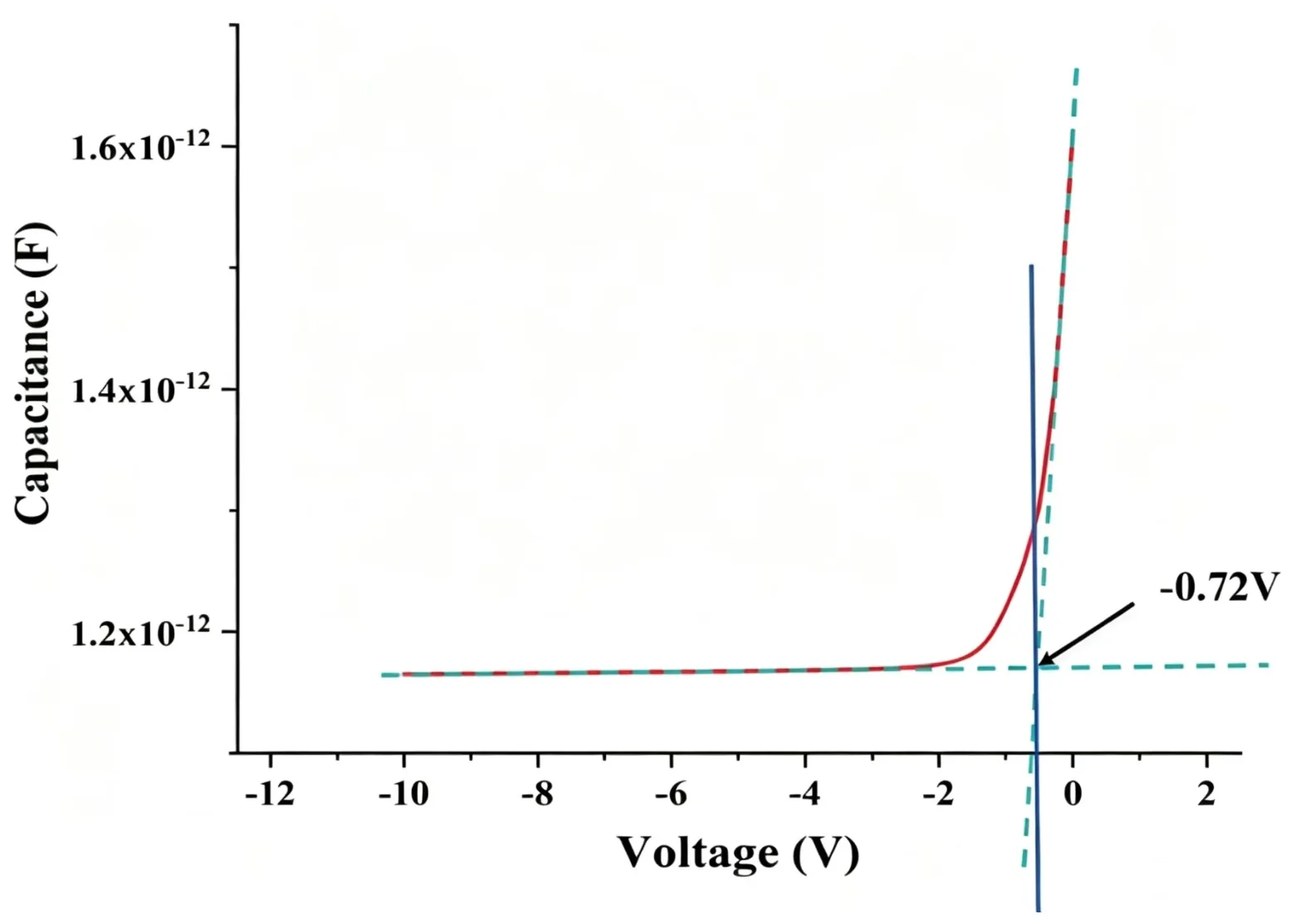
Capacitance–voltage curve of the detector under non-irradiated conditions.

Figure 8 shows the C-V characteristic curve of the device. As the reverse bias increases, the capacitance tends to flatten out, indicating that the device has entered the full-depletion state. The full-depletion voltage can be determined to be approximately −0.72 V through the intersection of the two tangent lines. This result indicates that only a relatively low reverse bias is required to achieve full depletion under non-irradiated conditions.

**Figure 9:**
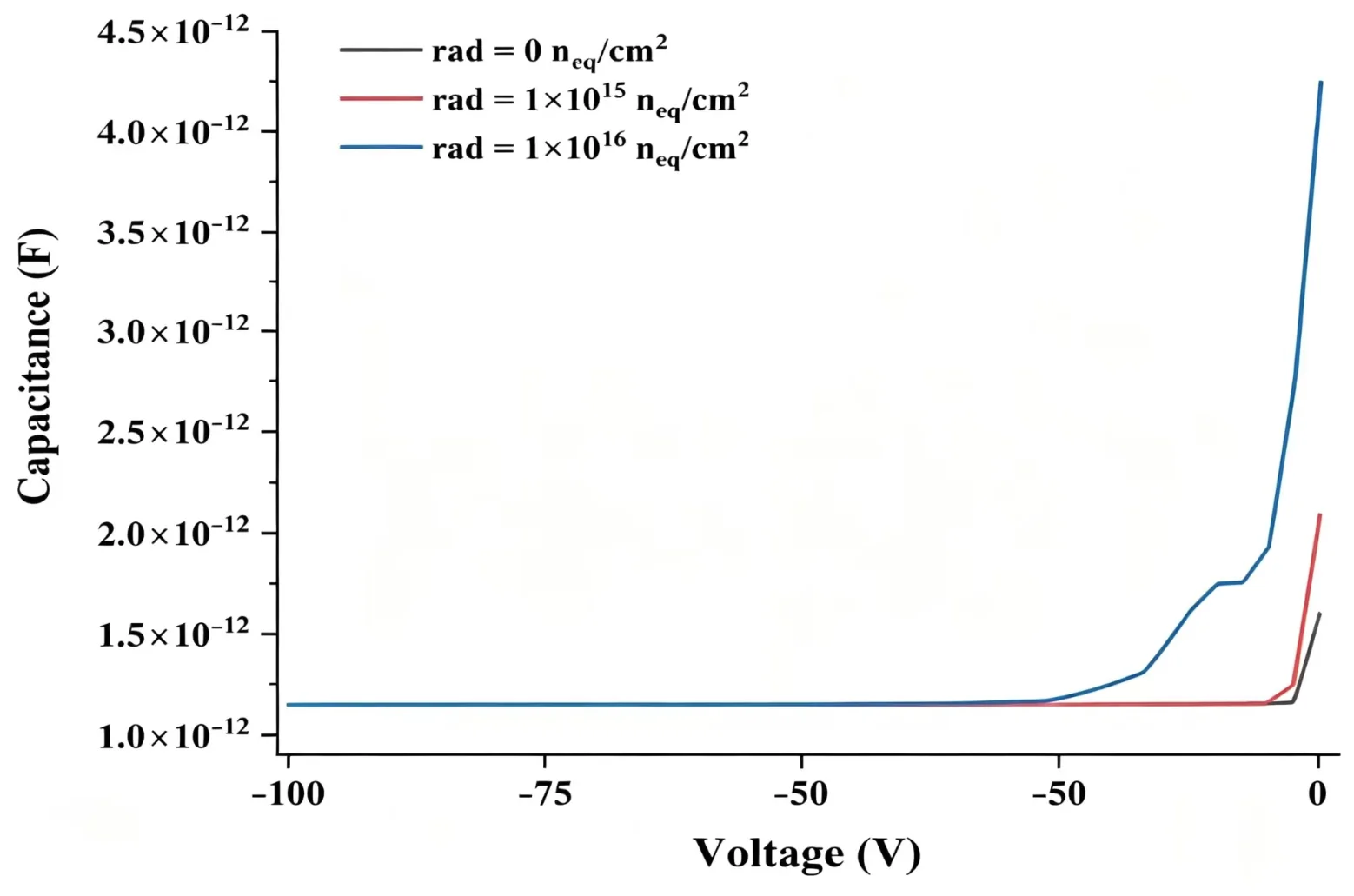
Capacitance–voltage curves of the detector under different radiation conditions.

Figure 9 shows the C-V characteristic curves of the detector under different neutron-equivalent irradiation fluences (0, 1 × 10^15^, and 1 × 10^16^ n_eq_/cm^2^). As can be seen from the figure, in the reverse bias voltage range of −25 V to −100 V, all three curves tend to be flat and perfectly overlap, stabilizing at about 1.15 × 10^−12^ F. This indicates that, even after undergoing extremely high-dose irradiation of 1 × 10^16^ n_eq_/cm^2^, the detector can still achieve full depletion when sufficient bias is applied, and its geometric capacitance remains intact [26]. For the un-irradiated (0 n_eq_/cm^2^) and low-fluence (1 × 10^15^ n_eq_/cm^2^) devices, the full-depletion voltage is extremely low, less than 1 V. When the irradiation fluence reaches 1 × 10^16^ n_eq_/cm^2^, the absolute value of the depletion voltage increases significantly, and the inflection point of the curve shifts leftwards to around −25 V. This phenomenon is mainly attributed to the fact that strong irradiation introduces a large number of deep-level defects into the silicon bulk region, changing the effective doping concentration (N_eff_) of the bulk, which causes the device to require a higher reverse bias voltage to achieve the full depletion of the bulk region. When the irradiation fluence reaches 1 × 10^16^ n_eq_/cm^2^, the magnitude of the full-depletion voltage increases to approximately 25 V. This shift can be attributed to radiation-induced deep-level defects and the resulting change in the effective space-charge concentration. Nevertheless, the detector remains fully depletable within the investigated bias range. These results demonstrate favorable depletion characteristics under the radiation damage model adopted in this work. However, the depletion voltage alone is insufficient to establish low-power operation or comprehensive radiation hardness, which would additionally require the evaluation of the leakage current, breakdown behavior, power dissipation, and charge trapping.

### 3.5. Transient Current Response and Charge Collection Dynamics

**Figure 10:**
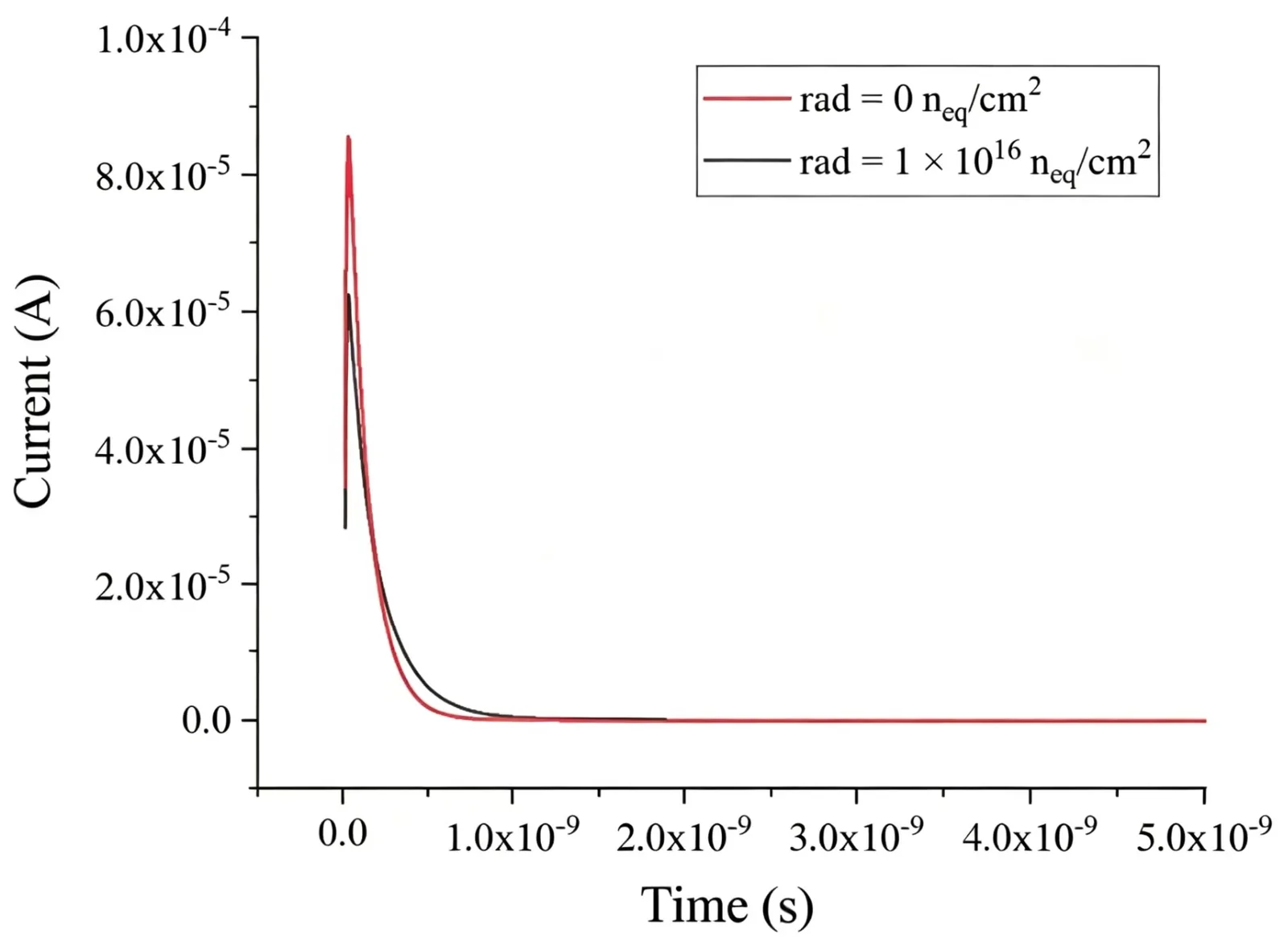
Simulated transient current pulses induced by particle incidence before and after extreme irradiation (1 × 10^16^ neq/cm^2^).

To directly investigate the charge collection dynamics and verify the radiation tolerance of the proposed interleaved trench structure, transient simulations under particle incidence were performed using Sentaurus TCAD. A minimum ionizing particle (MIP) track was simulated, perpendicularly penetrating through the active silicon bulk to generate electron–hole pairs along its trajectory. The transient induced current pulses on the readout electrode under an operating reverse bias of −25 V before and after irradiation are presented in Figure 10.

As shown in Figure 10, the un-irradiated detector (rad = 0 neq/cm^2^) exhibits an ultra-fast transient response characterized by a steep rising edge and a peak current reaching approximately 8.6 × 10^−5^ A. The induced current rapidly returns to the baseline, with the total charge collection time being less than 0.8 ns. This sub-nanosecond response is primarily attributed to the short carrier drift distance (20 μm inter-trench spacing) and the highly uniform lateral electric field, which enables the generated carriers to quickly reach saturation drift velocities.

After enduring extreme neutron-equivalent fluence of 1 × 10^16^ neq/cm^2^, the peak current slightly decreases to approximately 6.2 × 10^−5^ A, while the charge collection time remains well within 1.0 ns. The modest reduction in peak amplitude and the slight tailing effect are caused by radiation-induced deep-level defects, which trap free carriers and reduce carrier effective lifetimes. Despite the radiation-induced reduction in peak current and the slight increase in collection time, the proposed detector retains a clear transient response under the radiation damage conditions considered in this work. The total collected charge was obtained by integrating the transient current pulse according to

$$Q_{col}=\int_{t_{0}}^{t_{1}}I\left(t\right)dt$$

The integration of the simulated current pulses yields a collected charge of approximately 11.89 fC for the non-irradiated detector and approximately 11.90 fC after neutron-equivalent fluence of 1 × 10^16^ neq/cm^2^. The difference between the two values is less than approximately 0.1%, indicating that the total collected charge remains essentially unchanged within the numerical accuracy of the simulation, despite the irradiation-induced reduction in peak current and slight broadening of the transient pulse.

### 3.6. Performance Comparison with the Reference 3D Trench Electrode Detector

To further evaluate the characteristics of the proposed interleaved trench detector, a quantitative literature-based comparison was performed with the ultra-fast 3D trench electrode detector reported by Liu et al. [19]. The two detectors employ similar doping levels, with bulk and heavily doped electrode concentrations of approximately 1 × 10^12^ cm^−3^ and 1 × 10^19^ cm^−3^, respectively. However, their geometrical dimensions and electrode topologies are different. The reference detector consists of a central p^+^ column surrounded by an n^+^ trench in a 200 μm thick silicon substrate, with electrode spacings of 5 and 10 μm [19]. In contrast, the proposed detector employs interleaved n^+^ and p^+^ trench electrodes within a 120 × 120 μm^2^ detector cell with a thickness of 300 μm and inter-trench spacing of 20 μm. Therefore, the following comparison is intended to highlight the different performance characteristics of the two designs, rather than to represent a strictly controlled comparison under identical geometrical conditions.

A major structural difference between the two detectors is the geometry of the mechanically retained silicon region. In the reference detector, the 180 μm deep trench leaves an approximately 20 μm thick unetched silicon layer at the bottom of the 200 μm thick substrate. This region was reported to exhibit a relatively weak electric field because the trench electrode does not extend through the full detector thickness [19]. In the proposed detector, the mechanically retained silicon is instead limited to an approximately 1 μm wide support region on each lateral side. As demonstrated by the electric field distributions in Section 3.2, the low-field region on the investigated mid-plane is substantially reduced as the bias approaches the full-depletion condition. This structural arrangement is therefore expected to reduce the low-field volume associated with electrode termination while maintaining the mechanical integrity required during fabrication. Nevertheless, a full three-dimensional field analysis is still required to quantitatively determine the remaining low-field volume throughout the complete detector.

The two structures also exhibit different depletion characteristics. Liu et al. reported full-depletion voltages of approximately 1.5 V and 2.5 V for electrode spacings of 5 μm and 10 μm, respectively [19]. In comparison, the carrier concentration results indicate full depletion at approximately 0.7 V, while the C-V analysis yields a more precisely extracted full-depletion voltage of approximately 0.72 V. Notably, this low depletion voltage is obtained despite the larger 20 μm inter-trench spacing of the proposed structure. The result suggests that the interleaved arrangement of the n^+^ and p^+^ trenches provides efficient lateral depletion of the active silicon volume. However, because the substrate polarity, detector thickness, electrode spacing, and electrode geometry differ between the two designs, the difference in depletion voltage cannot be attributed exclusively to the interleaved topology.

With respect to the transient response, the reference detector was specifically optimized for extremely short carrier drift distances and exhibited response times in the tens-to-hundreds-of-picoseconds range. The proposed detector has a larger inter-trench spacing of 20 μm and therefore does not aim to exceed the timing performance of the ultra-fast reference structure. Nevertheless, the transient results presented in Section 3.5 show that the proposed detector maintains a sub-nanosecond charge collection response, with a collection time below approximately 0.8 ns before irradiation and remaining below approximately 1.0 ns after fluence of 1 × 10^16^ n_eq_/cm^2^. These results indicate that fast carrier collection can still be achieved in the proposed geometry despite its larger electrode spacing and greater detector thickness.

The larger detector thickness also provides a potentially larger active volume for particle-induced charge generation. The proposed detector has a thickness of 300 μm, compared with the 200 μm substrate thickness of the reference detector [19]. For a normally incident minimum ionizing particle, a larger active silicon thickness can, in principle, generate a larger amount of electron–hole charge, provided that the active volume is fully depleted and the generated carriers are efficiently collected. In the present transient simulation, the unirradiated detector produces a peak induced current of approximately 8.6 × 10^−5^ A. However, because the two studies employ different geometries, transient simulation conditions, and readout configurations, a direct quantitative comparison of the signal amplitude or signal-to-noise ratio is not performed here.

Under irradiation, the proposed detector also maintains favorable depletion and transient characteristics within the radiation damage model adopted in this work. At the neutron-equivalent fluence of 1 × 10^16^ n_eq_/cm^2^, the simulated full-depletion voltage increases to approximately −25 V, while the transient charge collection time remains below approximately 1.0 ns. These results indicate that the short lateral carrier drift distance provided by the interleaved trenches can help to limit the effect of radiation-induced trapping on the transient response. However, because the radiation damage models and simulation conditions of the two studies are not identical, these results should be interpreted as evidence of the radiation-tolerant behavior of the proposed detector rather than as proof of superior radiation hardness relative to the reference detector.

Overall, the proposed interleaved trench detector represents a different optimization strategy from the ultra-fast reference detector. Rather than minimizing the electrode spacing to achieve the shortest possible response time, the present design combines a larger 120 × 120 μm^2^ detector cell and 300 μm active thickness with an interleaved trench electrode topology, a low full-depletion voltage, reduced mechanically retained silicon regions, and sub-nanosecond charge collection. The comparison therefore suggests that the proposed structure provides a promising compromise among the depletion voltage, electric field distribution, detector thickness, transient response, and fabrication-related mechanical support. A strictly quantitative assessment of the intrinsic advantages of the interleaved topology will require future simulations in which the reference and proposed detectors are evaluated using identical dimensions, electrode spacings, physical models, boundary conditions, and radiation damage parameters.

## 4. Conclusions

In this work, a novel 3D silicon detector based on interleaved n^+^ and p^+^ trench electrodes was proposed and investigated using Sentaurus TCAD simulations. The proposed geometry differs from the reference 3D trench detector by replacing the conventional column–trench configuration with interleaved trench electrodes and by retaining only an approximately 1 μm wide unetched silicon support region on each lateral side. This design preserves the mechanical connection of the detector body during trench etching while reducing the supporting silicon region associated with weak electric fields near trench terminations.

The simulation results demonstrate the favorable electrostatic and charge transport characteristics of the proposed structure. As the reverse bias increases, the electric field distribution on the investigated mid-plane becomes more uniform and the low-field region is substantially reduced. The carrier concentration analysis indicates that the detector approaches full depletion at approximately 0.7 V, in agreement with the full-depletion voltage of approximately 0.72 V extracted from the C-V characteristics. Transient simulations further demonstrate that the detector achieves an ultra-fast sub-nanosecond charge collection response, with a total collection time below 0.8 ns before irradiation, which is directly enabled by the short lateral drift distance (20 μm) and the uniform high-field distribution.

To investigate the post-irradiation performance under fluence of 1 × 10^16^ n_eq_/cm^2^, a reverse bias of −25 V was selected for the simulations, which is sufficient to overcome the radiation-induced depletion voltage increase and maintain fast charge collection, while the charge collection time remains below approximately 1.0 ns. These results verify that the interleaved trench configuration effectively suppresses carrier trapping and mitigates low-field dead zones, ensuring stable full-depletion capabilities and fast charge collection even after extreme fluence of 1 × 10^16^ n_eq_/cm^2^.

The comparison with the reference ultra-fast 3D trench detector further shows that the proposed structure represents a different design trade-off. Although the two detectors differ in geometry, dimensions, substrate type, and electrode spacing, and this work therefore cannot be regarded as a strictly controlled one-to-one comparison, the proposed detector combines a low depletion voltage, reduced mechanically retained silicon regions, a 300 μm detector thickness, and sub-nanosecond charge collection. These results support the feasibility of the interleaved trench concept as a promising 3D detector architecture. Future work will focus on full-volume three-dimensional electric field analysis, the systematic optimization of the geometrical parameters, multi-pixel array simulations, and the experimental fabrication and characterization of the proposed detector.

## Data Availability

The data presented in this study are available from the corresponding author upon reasonable request.
